# Overexpression of FGFR2 in Mandibular Ameloblastoma Is Potentially Associated with Gene Amplification and Deletion

**DOI:** 10.3390/ijms27083443

**Published:** 2026-04-12

**Authors:** Nattanit Boonsong, Nakarin Kitkumthorn, Puangwan Lapthanasupkul, Kittipong Laosuwan, Wacharaporn Thosaporn, Jutamad Makyoo, Anak Iamaroon

**Affiliations:** 1 PhD Program in Oral Science, Faculty of Dentistry, Chiang Mai University, Under the CMU Presidential Scholarship, Chiang Mai 50200, Thailand; nattanit_boon@cmu.ac.th; 2 Faculty of Dentistry, Burapha University, Chonburi 20131, Thailand; nakarinkit@gmail.com; 3 Department of Oral and Maxillofacial Pathology, Faculty of Dentistry, Mahidol University, Bangkok 10400, Thailand; puangwan.lap@mahidol.ac.th; 4 Department of Oral Biology and Diagnostic Sciences, Faculty of Dentistry, Chiang Mai University, Chiang Mai 50200, Thailand; kittipong.l@cmu.ac.th; 5 Faculty of Dentistry, Nation University, Chiang Mai 50210, Thailand; wacharaporn_tho@nation.ac.th; 6 Department of Pathology, Faculty of Medicine, Chulalongkorn University, Bangkok 10330, Thailand; jutamad.mk27@gmail.com

**Keywords:** ameloblastoma, FGFR2, gene amplification, gene deletion, gene expression

## Abstract

Ameloblastoma (AM) is a common locally invasive benign odontogenic tumor in Asian populations. Although *fibroblast growth factor receptor 2* (*FGFR2*) mutations have been reported in AM, *FGFR2* amplification, the predominant form of *FGFR2* aberration in human cancers, remains unexplored. This study aimed to evaluate FGFR2 protein expression, *FGFR2* gene copy number variations, and their associations with demographic and clinico-radio-pathological parameters in mandibular AM. Eighty-seven cases of mandibular AM and 10 dental follicle (DF) specimens were included. FGFR2 protein expression was assessed by immunohistochemistry, and gene copy number variations were analyzed using the quantitative real-time polymerase chain reaction (qPCR) technique. Clinical data, including age, gender, tumor size, radiographic features, histological subtype, and recurrence history, were examined for associations with FGFR2 alterations. FGFR2 protein overexpression was observed in 95.4% of AM cases and was not significantly associated with demographic or clinico-radio-pathological variables. *FGFR2* gene amplification was detected in 52.5% of cases, while 8.2% showed gene deletion. Notably, 50.8% of cases exhibited concurrent *FGFR2* amplification and overexpression, and all cases with *FGFR2* gene deletion also demonstrated FGFR2 overexpression. These findings suggest that *FGFR2* gene amplification and deletion may contribute to FGFR2 overexpression and play a significant role in the molecular pathogenesis of mandibular AM.

## 1. Introduction

Ameloblastoma (AM) is the most common benign odontogenic tumor of the jaw, characterized by its slow growth yet pronounced local invasiveness [1,2]. Epidemiological data from recent studies indicate a peak incidence in between the third and fifth decades, with a slight weighting towards males of approximately 53% [3,4]. While AM occurs globally, its incidence is notably higher among Asians compared to other populations [2], reflecting variable genetic aberration among different ethnic groups.

Clinically, approximately 80–91% of AM cases are localized to the mandible, predominantly affecting the posterior region [3,4]. AM is histologically diverse, with the follicular and plexiform patterns being the most prevalent. Specifically, the solid/multicystic subtype is associated with a significantly higher recurrence rate, often exceeding 40% when treated conservatively, compared to the unicystic variant [3]. Consequently, the management of AM remains a significant clinical challenge. While wide margin resection (ideally 1.0–1.5 cm) is the established gold standard for minimizing recurrence, recent multicenter studies emphasize a balanced surgical approach. This strategy seeks to mitigate the risk of recurrence while prioritizing functional and aesthetic outcomes through immediate reconstruction, particularly in light of the psychological impact of facial disfigurement in younger patients [3,5]. In recent decades, considerable attention has been directed towards elucidating the molecular mechanisms of AM pathogenesis. Evidence indicates dysregulation of the mitogen-activated protein kinase (MAPK) pathway as a key driver [1]. A prevalent mutation in mandibular AM, well-established in the MAPK pathway, is the *rapidly accelerated fibrosarcoma* B-type (*BRAF*) mutation [6]. Furthermore, mutations within *rat sarcoma virus* (*RAS*) and *fibroblast growth factor receptor 2* (*FGFR2*) genes also contribute to the dysregulated signaling cascade [7].

FGFR2, a member of the fibroblast growth factor receptor family encoded by the *FGFR2* gene on chromosome 10 (10q26.13), is crucial for the activation of various signaling pathways, including the MAPK pathway [8]. Activation of FGFR2 initiates downstream signaling events that regulate cellular growth, proliferation, and survival; thus, genetic alterations in FGFR2 significantly contribute to pathway dysregulation [8,9]. These alterations contribute to the initiation and progression of various diseases, particularly neoplasms [10]. Aberrations in the *FGFR2* gene have been extensively documented in various malignancies such as endometrial, gastric, breast, and head and neck cancers [10,11,12,13]. The predominant form of FGFR2 aberrations is gene amplification, accounting for 49%, followed by mutations at 18%, fusions at 15%, and other rare alterations representing 18% [9]. Furthermore, recent data have suggested a role for FGFR2 aberrations in AM pathogenesis, with mutations detected in 6–18% of cases [6,14].

Despite emerging evidence of FGFR2 mutations in AM, the current literature has given insufficient attention to gene copy number variations. Furthermore, the specific relationship between *FGFR2* copy number alterations, protein overexpression, and clinico-radio-pathological characteristics remains unexplored. A preliminary study by our group identified FGFR2 amplification in 26.7% of AM samples, suggesting a potential association with protein overexpression [15]. Therefore, the present study was designed to evaluate FGFR2 at both the genomic and protein levels to clarify its role in the molecular landscape of mandibular AM.

Building upon the existing literature, this study aimed to investigate FGFR2 expression and gene copy number variations in a cohort of patients with mandibular AM. Our objectives were to: (1) examine FGFR2 expression levels and the prevalence of *FGFR2* gene copy number variations, and (2) explore potential associations between FGFR2 expression, *FGFR2* gene copy number variations, and demographic and clinico-radio-pathological parameters in patients with mandibular AM. The null hypothesis was that FGFR2 expression and gene copy number variations were not significantly altered in mandibular AM and were not associated with demographic or clinico-radio-pathological characteristics. Through this analysis, we intended to provide novel insights into the molecular landscape of mandibular AM and potentially identify FGFR2 as a therapeutic target.

## 2. Results

### 2.1. Overexpression of FGFR2

**Figure 1:**
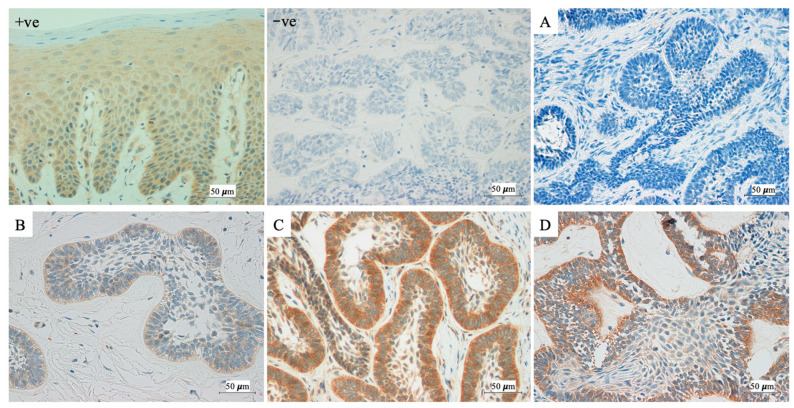
Immunostaining of FGFR2 in positive control (+ve), negative control (−ve), and representative cases of ameloblastoma (AM) (**A**–**D**); (**A**) negative immunoreaction of FGFR2 in AM, (**B**) low-level overexpression of FGFR2 in AM, (**C**) high-level overexpression of FGFR2 in a follicular variant of AM, (**D**) high-level overexpression of FGFR2 in a plexiform variant of AM (immunohistochemistry, original magnification ×400).

Immunohistochemical analysis showed that all DF samples were FGFR2 negatively immunostained. Compared to DF, 95.4% of mandibular AM exhibited FGFR2 overexpression, with 29.9% showing low-level and 65.5% high-level expression. Only four mandibular AM exhibited negative FGFR2 immunoactivity. The localization of FGFR2 expression was observed within the cytoplasm of both ameloblast-like and stellate-reticulum-like cells. Notably, the intensity of cytoplasmic immunostaining of FGFR2 was stronger in ameloblast-like cells than in stellate-reticulum-like cells. FGFR2 expressions in AM are displayed in Figure 1.

The associations between FGFR2 expression and demographic data and clinico-radio-pathological features are shown in Table 1. However, there was no significant association between FGFR2 expression and all parameters studied.

### 2.2. FGFR2 Gene Copy Number Variations

**Figure 2:**
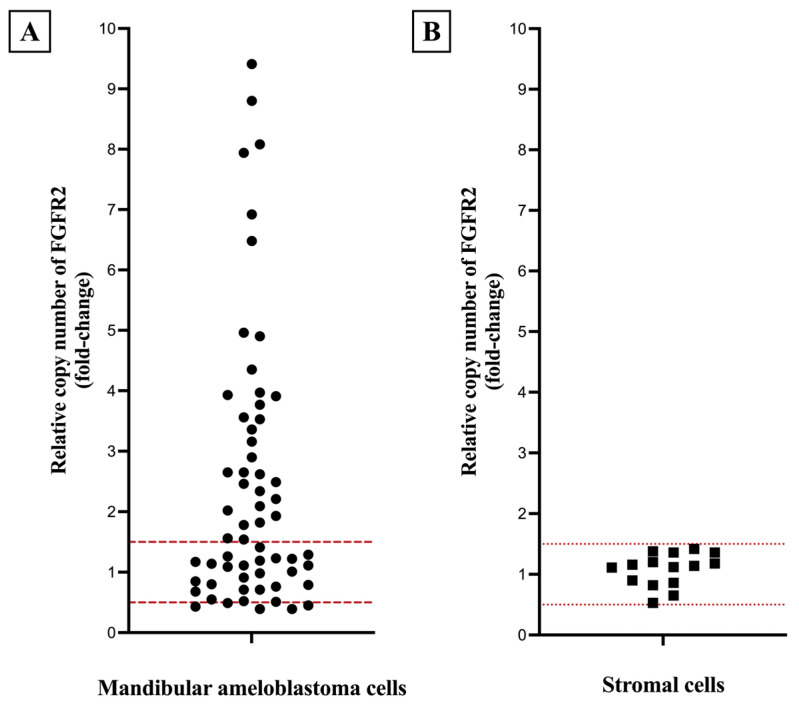
Relative copy numbers of *FGFR2* gene (fold-change or 2^−ΔΔCT^ value); (**A**) in all cases of mandibular ameloblastoma cells, and (**B**) in stromal cells isolated from mandibular ameloblastoma with amplified or deleted *FGFR2*. The red dashed lines indicate the range of normal *FGFR2* gene copy number.

Analysis of *FGFR2* gene copy number variations was performed on 61 out of 87 mandibular AM, with 26 cases excluded due to insufficient genomic DNA quality. *FGFR2* amplification was identified in 52.5% (32 out of 61 cases) of the analyzed mandibular AM. *FGFR2* deletion was observed in 8.2% (5 out of 61 cases). The remaining AM and all DF samples exhibited normal *FGFR2* copy numbers. The relative copy number (fold-change) of *FGFR2* gene in mandibular AM compared to DF is represented in Figure 2A. Furthermore, stromal cells isolated from 15 cases with amplified or deleted *FGFR2* in the tumor cells demonstrated a normal *FGFR2* copy number (Figure 2B). This finding confirms that *FGFR2* was truly amplified or deleted in the tumor cells.

The details of the associations between *FGFR2* copy number variations and patients’ demographic data, as well as clinico-radio-pathological features, are presented comprehensively in Table 2. A significant association was observed between *FGFR2* copy number variations and the radiographic features of AM (*p* = 0.028). Specifically, mandibular AM exhibiting unilocular radiolucency was more likely to have *FGFR2* gene amplification compared to those with a multilocular radiographic presentation (OR = 2.35, 95% CI: 0.76–7.34).

### 2.3. Association Between FGFR2 Expression and FGFR2 Gene Copy Number Variations

**Figure 3:**
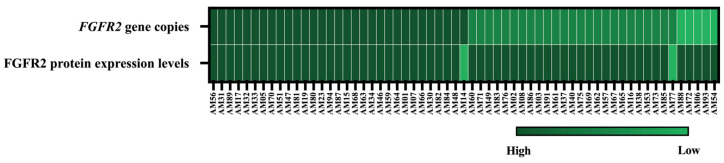
A heatmap of *FGFR2* gene copies and FGFR2 protein expression levels in all cases of mandibular ameloblastoma.

A notable association was observed between FGFR2 protein expression and *FGFR2* gene amplification and deletion. Considering all studied mandibular AM cases, 50.8% exhibited both FGFR2 overexpression and gene amplification. Moreover, FGFR2 overexpression was detected in all AM cases with *FGFR2* gene deletion (Figure 3 and Table 3).

## 3. Discussion

AM is characterized as a benign neoplasm with the potential for local invasion [1,2]. Resection with wide margins is the gold standard for treatment to minimize recurrences. However, the functional impairments and esthetic deformities following extensive surgery can significantly impact the patient’s quality of life [5]. Recognizing this challenge, several studies have advanced our understanding of the underlying molecular mechanisms driving AM, thereby raising the promising prospect of utilizing molecularly targeted therapies as a less morbid treatment approach [16].

The present study investigated the *FGFR2* gene copy number variations and FGFR2 expression in mandibular AM, aiming to elucidate its potential role in the pathogenesis of mandibular AM. Our findings demonstrated a high prevalence of *FGFR2* protein overexpression (95.4%) in mandibular AM. These results were in line with two previous immunohistochemical studies. One study demonstrated a strong FGFR2 expression in 30 out of 32 cases (93.75%) of AM [17]. Another study reported FGFR2 immunoreactivity in all AM specimens (100%), with a further observation of more intense staining in recurrent lesions compared to the primary tumors [18]. Taken together, these results suggest a potential role for FGFR2 overexpression in driving AM pathogenesis.

Furthermore, *FGFR2* gene amplification and deletion were detected in 52.5% and 8.2% of all AM cases, respectively. Although *FGFR2* gene amplification and protein overexpression have been reported in several neoplasms, including endometrial, breast, head and neck, and gastric cancers [10,11,12,13], to the best of our knowledge, the study on FGFR2 overexpression together with *FGFR2* gene amplification and deletion has not been evaluated before in AM. Our preliminary study, involved 30 samples of maxillary (three cases) and mandibular AM (27 cases), showed that 26.7% of 30 samples with *FGFR2* gene amplification were also associated with FGFR2 overexpression [15]. In this current study, we focused only on cases of mandibular AM, provided that 27 samples of mandibular AM from a previous study were used together with 37 new cases of mandibular AM (64 cases in sum). The present study confirmed our previous study that *FGFR2* amplification at a higher frequency and we newly identified *FGFR2* deletion in the current study. These findings underscore the significance of both gene amplification and deletion in mandibular AM. Moreover, FGFR2 overexpression with *FGFR2* gene amplification was observed in 50.8% of all AM cases. This observation implies that gene amplification could be a primary driver for increased protein expression.

In addition, our results demonstrated that all AM cases with *FGFR2* gene deletion also showed FGFR2 overexpression, indicating that FGFR2 dysregulation, in either mechanism (deletion or amplification), may contribute to the pathogenesis of mandibular AM. In the present study, *FGFR2* deletion was defined based on reduced relative gene copy number detected by qPCR at the analyzed *FGFR2* locus. Because the qPCR assay targeted only a limited region of the *FGFR2* gene, this approach cannot distinguish between whole-gene copy number loss, partial gene deletions, or other structural genomic alterations affecting the *FGFR2* locus. Therefore, the precise genomic nature of the detected deletions remains unclear.

A recent similar study in cholangiocarcinoma revealed *FGFR2* deletion, particularly in-frame deletions within the extracellular domain of the receptor, may alter ligand-binding specificity or promote ligand-independent receptor dimerization [19]. Furthermore, a recent study demonstrated that partial deletions of exon 18 of *FGFR2* result in a highly potent, clinically actionable oncogene by removing auto-inhibitory domains and increasing protein stability [20]. These alterations, in turn, could lead to the constitutive activation of FGFR2 signaling and oncogenic transformation. Although these mechanisms have been described in other tumor type, their relevance to mandibular AM remains to be clarified. Therefore, these findings may suggest potential mechanisms through which FGFR2 deletion could contribute to tumorigenesis in mandibular AM, but this interpretation should be considered cautiously. Alternatively, other unknown regulatory mechanisms may also be involved in cases of FGFR2 overexpression with gene deletion. Further investigations are required to better understand this phenomenon.

Moreover, our findings suggest that patients with *FGFR2*-amplified or *FGFR2*-deleted mandibular AM may benefit from targeted therapies directed against FGFR2. While this clinical role is currently underexplored and lacks validation through in vitro or in silico models, two case reports using FGFR inhibitors, lenvatinib and erdafitinib, in patients with *FGFR2*-mutant AM have shown favorable clinical responses with marked decreases in tumor size. These preliminary observations suggest that FGFR inhibitors could be considered as a possible treatment option for patients with AM harboring *FGFR2* gene alterations who are unwilling or unable to undergo standard care [21,22]. However, further studies are warranted to confirm the objective therapeutic potential of these genomic alterations.

Notably, a significant association was observed between *FGFR2* gene amplification and radiographic features. Specifically, AM cases with unilocular radiolucency were more likely to exhibit *FGFR2* gene amplification compared to those with multilocular radiolucency. This finding may explain that *FGFR2* amplification may promote early tumor growth, with subsequent molecular events potentially leading to the development of a multilocular architecture. Nevertheless, specific studies focusing on *FGFR2* amplification in early- versus late-stage AM lesions are currently lacking. Therefore, further investigation is required to elucidate the role of FGFR2 in the progression of AM.

In the present study, a minority of mandibular AM cases (37.7%) showed FGFR2 protein overexpression without gene amplification or deletion. This finding indicates that mechanisms other than gene amplification and deletion, particularly *FGFR2* point mutations (e.g., missense or nonsense mutations), may also contribute to protein overexpression. In fact, previous studies have reported *FGFR2* point mutations in 6–18% of AM cases [6,14]. Moreover, *FGFR2* point mutations at S252W, P253R, and N549K have been identified in endometrial, lung, and gastric cancers. These mutations led to increased ligand affinity, altered ligand specificity, ligand-independent activation of the receptor [23], and probably overexpression of FGFR2 protein as a consequence. Nonetheless, the association between FGFR2 point mutations and protein overexpression in AM has not yet been explored. Taken together, to address these limitations, we are currently conducting genetic sequencing to identify the plausibility of *FGFR2* point mutations and their role in the pathogenesis of AM.

Additionally, an important factor in interpreting the significance of *FGFR2* dysregulation is its relationship with the other established oncogenic drivers in AM. In fact, we recently reported that *BRAF* V600E mutation was the most frequent gene alteration in AM [24]. Although the present study did not evaluate *BRAF* V600E or other mutations, it is plausible that a considerable proportion of our cohort also harbors *BRAF* V600E mutation. A co-mutation of *FGFR2* and *BRAF* V600E in AM may lead to novel strategies for targeted therapies. Future studies on these interesting issues will elucidate a better understanding of the molecular pathogenesis of AM.

One of the limitations of the present study is that data on *FGFR2* gene amplification and deletion were derived exclusively from the qPCR technique. Quantitative PCR is widely used for assessing gene copy number variations because it requires relatively small amounts of DNA, is compatible with archival FFPE tissues, and allows sensitive detection of relative changes in gene dosage. However, although qPCR technique provides valuable information regarding potential alterations in gene dosage, it is an indirect approach for evaluating genomic copy number variation. Specifically, our utilized cutoffs of >1.5 for amplification and <0.5 for deletion represent relative fold-changes in DNA concentration rather than direct physical counts of gene loci. Direct assessments of gene dosage, such as fluorescence in situ hybridization (FISH) or genetic sequencing, are, therefore, considered confirmatory methods. We have aimed to include these methods in our future studies on *FGFR2* gene analyses in AM.

A methodological limitation of the present study was the use of tumor-associated fibrous stroma as the comparator for copy number analysis rather than normal tissue, such as matched peripheral blood or uninvolved tissue. While the use of tumor-associated fibrous stroma as the comparator provides a reliable internal control, it entails two considerations. First, stromal tissue derived from the tumor microenvironment may not truly represent normal tissue due to potential molecular shifts induced by paracrine signaling from adjacent neoplastic cells. Second, despite thorough microdissection, the possibility of minor contamination with neoplastic cells cannot be completely excluded. Collectively, the copy number variation data in the present study should, therefore, be interpreted with caution.

Another limitation of the present study is that gene copy number analysis could be successfully performed in only 61 of the 87 AM samples due to insufficient DNA quality obtained from archival FFPE tissues. The exclusion of these cases may have reduced the statistical power of the copy number analysis and could potentially affect the representativeness of the study population. Although the remaining sample size was still adequate for exploratory analysis, the reduced number of evaluable cases may limit the strength of the observed associations. Therefore, the findings related to *FGFR2* gene copy number alterations should be interpreted with caution. Future studies including larger sample sizes with optimal DNA quality will be helpful to further validate these observations.

In addition, the relatively low number of patients who experienced disease recurrence represents another important limitation. Due to the retrospective study design, the number of relapsed cases was inherently constrained by the availability of archival records and the duration of follow-up, resulting in a small recurrence subgroup. This limited the statistical power and restricted further analyses aimed at evaluating the prognostic impact of FGFR2 alterations. Future prospective investigations with larger cohorts and standardized long-term follow-up are warranted to enable more comprehensive prognostic modeling and to clarify the clinical significance of FGFR2 dysregulation in mandibular AM.

## 4. Materials and Methods

### 4.1. Sample Collection

Eighty-seven formalin-fixed paraffin-embedded (FFPE) tissue samples of mandibular AM, without decalcification for appropriate immunohistochemical staining, and 10 FFPE tissue samples of dental follicle (DF), obtained from asymptomatic, unerupted, and non-inflamed embedded teeth, were collected from the archives of the Oral Pathology laboratories, Faculty of Dentistry, Chiang Mai University, from 2012 to 2022. The diagnosis of all cases was confirmed by experienced oral and maxillofacial pathologists in accordance with the 2017 World Health Organization (WHO) Classification of Head and Neck Tumours [25]. Demographic data and clinico-radio-pathological information, including patients’ sex, age at diagnosis, tumor sizes, radiographic characteristics, histopathological subtypes, and history of recurrence, were collected. This research was ethically approved by the Human Experimental Committee of the Faculty of Dentistry, Chiang Mai University (No. 32/2022). All procedures were performed in accordance with the committee guidelines and regulations.

### 4.2. Immunohistochemistry (IHC)

FFPE tissue blocks were cut into 3 μm thick sections for immunohistochemical staining with FGFR2 rabbit polyclonal primary antibody (ab10648, lot GR3214128-9, Abcam, Cambridge, MA, USA, dilution 1:2000) on the Ventana^®^ Benchmark Ultra automated slide stainer (Ventana Medical Systems, Tucson, AZ, USA) using a protocol previously described [26]. Positive (normal oral mucosa, reported to be positively immunostained for FGFR2 [27]) and negative (no primary antibody added) controls were included in each run.

For immunohistochemical analysis, two pathologists independently assessed the immunoreactivity scores for FGFR2 using the immunoreactive score (IRS) system [28] from five discrete representative areas at high magnification (×400). The IRS was evaluated based on two criteria: staining intensity (0 = none, 1 = weak, 2 = moderate, 3 = strong) and percentage of positive cells (0 = none, 1 = <10%, 2 = 10–50%, 3 = >50%). Scores were generated by multiplying staining intensity and positive cell percentage. A score of 0 indicated no expression, scores 1 to 5 indicated low expression, and scores greater than 5 indicated high expression. In case of any disagreement between the two pathologists, a consensus was reached.

### 4.3. Tissue Microdissection and Genomic DNA Extraction

Prior to genomic DNA extraction, tumor cells from each mandibular AM were isolated from surrounding connective tissue stroma using a manual microdissection, as previously described [29]. Sections containing less than 50% of neoplastic cells were deemed inadequate and excluded. In addition, sections in which the neoplastic area was limited (smaller than 0.5 cm^2^) to allow accurate and reliable microdissection were also excluded [30]. The dissected tissues were temporarily stored in phosphate-buffered saline (PBS) until DNA extraction. Following established protocols, the standard phenol-chloroform technique was used to extract genomic DNA [31]. The NanoDrop™ 2000 Spectrophotometer (Thermo Fisher Scientific, Wilmington, DE, USA) was employed to assess the extracted DNA’s concentration (260 nm absorbance) and purity (260/280 ratio). Finally, the DNA concentration was adjusted to 50 ng/μL and stored at below −20 °C for subsequent analyses.

### 4.4. Quantitative Real-Time Polymerase Chain Reaction (qPCR)

The copy number of *FGFR2* gene was assessed using SYBR^®^ Green-based qPCR assay with the CFX96 Touch Real-Time PCR Detection System and Bio-Rad CFX Maestro Software (version 1.1.4, Bio-Rad Laboratories, Hercules, CA, USA). To prepare the qPCR reaction, a total volume of 10 μL containing 50 ng/μL of extracted genomic DNA, specific forward and reverse primers, and Luna^®^ Universal qPCR Master Mix (New England Biolabs, Ipswich, MA, USA) was used. The primer sequences for the *FGFR2* gene were as follows: forward, 5′-TGGTTGACAGTTCTGCCAGG-3′, and reverse, 5′-TTATGCAAGGATAAAAGGGGCC-3′. As reference gene, the *human globin* (*HGB*) gene was determined with its primers as follows: forward, 5′-GTGCACCTGACTCCTGAGGAGA-3′, and reverse, 5′-CCTTGATACCAACCTGCCCAG-3′. Primers for *FGFR2* and the reference gene *HGB* were designed based on the NCBI GenBank accession numbers NG_012449 and U01317, respectively. Each reaction underwent an initial phase at 95 °C for 3 min, followed by 40 cycles of incubation at 95 °C for 10 s and 60 °C for 30 s. A negative control, containing sterile water instead of a DNA sample, was included in each run. All reactions were performed in duplicate and quantified based on the threshold cycle value (C_T_) using the comparative threshold cycle or 2^−ΔΔCT^ method [32]. FGFR2 copy number variation results were then expressed as fold-change (2^−ΔΔCT^ value). Based on the modified cutoffs criteria from a previous study [33], a gene was considered amplified when the 2^−ΔΔCT^ value was >1.5 and deleted when the 2^−ΔΔCT^ value was <0.5. Values between 0.5 and 1.5 were considered as unchanged.

To validate the results obtained from AM cells, fibrous stromal tissue, serving as a representative of normal cells, was collected from the same samples showing *FGFR2* amplification or deletion. Subsequently, the FGFR2 gene copy number within these normal cell populations was quantified using the aforementioned qPCR assay.

### 4.5. Statistical Analyses

The statistical analyses were conducted using IBM^®^ SPSS^®^ Statistics version 25 (IBM Corporation, Armonk, NY, USA). Associations between FGFR2 protein expression, *FGFR2* gene copy number variations, and patients’ demographic and clinico-radio-pathologic data were analyzed using Fisher’s exact test due to small subgroup sample sizes. A *p*-value < 0.05 was accepted as statistically significant. Logistic regression analysis was performed, where possible, to investigate the association between FGFR2 alterations and independent variables, including patients’ sex, age at diagnosis, tumor sizes, radiographic characteristics, histopathological subtypes, and history of recurrence. However, in some analyses, an odds ratio (OR) could not be reliably calculated due to zero counts in certain contingency table cells.

## 5. Conclusions

The present study was the first to demonstrate that FGFR2 was overexpressed in most cases of mandibular AM, and FGFR2 overexpression may be associated with FGFR2 gene copy number alterations, including amplification and deletion. These findings highlight that FGFR2 may represent an important molecular player in AM pathogenesis and underscore its potential as a therapeutic target. Further studies, particularly on point mutations of *FGFR2* gene, are warranted to fill the gap of the mechanism of FGFR2 overexpression in mandibular AM. While such investigations may eventually lead to targeted therapy for patients with mandibular AM, current evidence remains preliminary. Additionally, the studies on the correlation of *FGFR2* gene dosage and expression will also provide a better understanding of the molecular pathogenesis of AM.

## Figures and Tables

**Table 1:** The associations between FGFR2 expression and patients’ demographic data and clinico-radio-pathological features.

Variables	Total Number	FGFR2 Expression			
		No Expression	Overexpression	OR (95% CI) ^†^	*p*-Value *
	87 (100.0%)	4 (4.6%)	83 (95.4%)		
Sex					
Male	52 (59.8%)	4 (7.7%)	48 (92.3%)	-	0.145
Female	35 (40.2%)	0 (0.0%)	35 (100.0%)	-	
Age (years; mean = 34.84)					
<20	22 (25.3%)	0 (0.0%)	22 (100.0%)	-	0.568
≥20	65 (74.7%)	4 (6.2%)	61 (93.8%)	-	
Size (cm.)					
<3.5	22 (25.3%)	0 (0.0%)	22 (100.0%)	-	0.568
≥3.5	65 (74.7%)	4 (6.2%)	61 (93.8%)	-	
Radiograph					
Multilocular	51 (58.6%)	4 (7.8%)	47 (92.2%)	-	0.139
Unilocular	36 (41.4%)	0 (0.0%)	36 (100.0%)	-	
Histopathological subtype					
Follicular	16 (18.4%)	0 (0.0%)	16 (100.0%)	-	0.309
Plexiform	42 (48.3%)	1 (2.4%)	41 (97.6%)	1.89 (0.09–37.53)	
Others	29 (33.3%)	3 (10.3%)	26 (89.7%)	1 (reference)	
Recurrence					
Presence	13 (14.9%)	2 (15.4%)	11 (84.6%)	0.16 (0.01–2.90)	0.104
Absence	74 (85.1%)	2 (2.7%)	72 (97.3%)	1 (reference)	

* *p*-value less than 0.05 was considered significant. ^†^ Odds ratio (OR) could not be calculated for some comparisons due to zero counts in contingency table cells.

**Table 2:** The associations between *FGFR2* copy number variations and patients’ demographic data and clinico-radio-pathological features.

Variables		*FGFR2* Copy Number Variations			OR (95% CI) ^†^	
	Total Number	Deletion	Normal	Amplification		*p*-Value *
	61 (100.0%)	5 (8.2%)	24 (39.3%)	32 (52.5%)		
Sex						
Male	35 (57.4%)	3 (8.6%)	15 (42.9%)	17 (48.6%)	1 (reference)	
Female	26 (42.6%)	2 (7.7%)	9 (34.6%)	15 (57.7%)	1.33 (0.43–4.11)	0.853
Age (years)						
<20	22 (36.1%)	2 (9.1%)	9 (40.9%)	11 (50.0%)	1 (reference)	
≥20	39 (63.9%)	3 (7.7%)	15 (38.5%)	21 (53.8%)	1.64 (0.51–5.27)	1.000
Size (cm)						
<3.5	17 (27.9%)	2 (11.8%)	4 (23.5%)	11 (64.7%)	1 (reference)	
≥3.5	44 (72.1%)	3 (6.8%)	20 (45.5%)	21 (47.7%)	0.57 (0.17–1.89)	0.265
Radiograph						
Multilocular	34 (55.7%)	1 (2.9%)	18 (52.9%)	15 (44.1%)	1 (reference)	
Unilocular	27 (44.3%)	4 (13.3%)	6 (23.3%)	17 (63.3%)	2.35 (0.76–7.34)	0.028 *
Histopathological subtype						
Follicular	8 (13.1%)	2 (25.0%)	2 (25.0%)	4 (50.0%)	1.19 (0.20–7.06)	
Plexiform	34 (55.7%)	1 (2.9%)	14 (41.2%)	19 (55.9%)	1.49 (0.44–5.07)	0.317
Others	19 (31.1%)	2 (10.5%)	8 (42.1%)	9 (47.4%)	1 (reference)	
Recurrence						
Presence	6 (9.8%)	2 (33.3%)	1 (16.7%)	3 (50.0%)	0.49 (0.07–3.47)	
Absence	55 (90.2%)	3 (5.5%)	23 (41.8%)	29 (52.7%)	1 (reference)	0.087

* *p*-value less than 0.05 was considered significant. ^†^ Odds ratios were calculated by logistic regression for amplification versus non-amplification.

**Table 3:** Association between *FGFR2* copy number variation and FGFR2 expression.

FGFR2 Expression		*FGFR2* Copy Number Variations		
	Total Number	Deletion	Normal	Amplification
	61 (100.0%)	5 (8.2%)	24 (39.3%)	32 (52.5%)
No expression	2 (3.3%)	0 (0.0%)	1 (50.0%)	1 (50.0%)
Overexpression	59 (96.7%)	5 (8.5%)	23 (39.0%)	31 (52.5%)

## Data Availability

The raw data supporting the conclusions of this article will be made available by the authors on request.

## Abbreviations

The following abbreviations are used in this manuscript:

AM	Ameloblastoma
BRAF	Rapidly accelerated fibrosarcoma B-type
C_T_	Threshold cycle value
DF	Dental follicle
FFPE	Formalin-fixed paraffin-embedded
FGFR2	Fibroblast growth factor receptor 2
HGB	Human globin
IHC	Immunohistochemistry
MAPK	Mitogen-activated protein kinase
PBS	Phosphate-buffered saline
qPCR	Quantitative real-time polymerase chain reaction
RAS	Rat sarcoma virus
WHO	World Health Organization
